# Breeding behavior analysis in a large captive colony of African penguins (*Spheniscus demersus*) and its implications for population management and conservation

**DOI:** 10.1038/s41598-024-54105-w

**Published:** 2024-02-13

**Authors:** Paweł Borecki, Joanna Rosenberger, Anna Mucha, Agnieszka Partyka

**Affiliations:** 1 Department of Reproduction and Clinic of Farm Animals, Wroclaw University of Environmental and Life Sciences, Pl. Grunwaldzki 49, 50-366 Wrocław, Poland; 2 Zoo Wrocław, Ul. Wróblewskiego 1-5, 51-618 Wrocław, Poland; 3 Division of Poultry Breeding, Institute of Animal Breeding, Wroclaw University of Environmental and Life Sciences, Ul. Chełmońskiego 38C, 51-630 Wrocław, Poland; 4 Department of Genetics, Wrocław University of Environmental and Life Sciences, Ul. Kożuchowska 7, 51-631 Wrocław, Poland

**Keywords:** Ecology, Population dynamics, Behavioural ecology, Animal physiology, Reproductive biology

## Abstract

The African penguin *Spheniscus demersus*, frequently housed in zoos, holds potential for future reintroduction efforts due to its declining wild population. This paper aims to explore various aspects of reproductive performance in African penguins within a large ex situ colony at Zoo Wrocław in Poland, covering 9 years of breeding behaviors. The analysis reveals parallels in colony growth and partner change patterns with those observed in the wild. Positive correlations were found between breeding success and pair-bond duration, with the increasing colony size influencing reproductive performance. Contrary to their wild counterparts, captive African penguins initiate breeding attempt and produce a fertilized egg at a younger age. However, successful breeding still requires gaining experience or forming pairs with more experienced partners. Our research indicates that providing captive African penguins with unlimited food resources and sufficient nesting space results in rapid colony growth. The increased colony size facilitates breeding behaviors that positively influence population dynamics, particularly through the maintenance of long-term pair bond relationships and the potential for partner changes when necessary or desirable to enhance breeding success. We present compelling case studies in pair fidelity, offering valuable insights and implications for the management of captive populations and conservation efforts.

## Introduction

There are 18 penguin species worldwide, all residing in the Southern Hemisphere. The African penguin (*Spheniscus demersus*) is unique to the African continent, specifically along the south-western coast of Namibia and South Africa^1^. Once abundant, its population has sharply declined from nearly 3 million in the early twentieth century to less than 30 thousand today, mainly due to human activities^2–7^. Historically depleted by guano harvesting and egg collection^2^, recent research indicates that the primary cause of the dramatic population decline in present days is diminishing food supplies^8,9^.

In 2010, the species was declared Endangered by International Union for Conservation of Nature and Natural Resources^10^. As part of Africa’s natural heritage, various conservation actions have been implemented to prevent further population decline. Much research has been conducted in situ, at breeding colonies, focusing on the species’ breeding patterns to gain insight into ways to protect those endangered birds^3,4,6,11–24^.

Not all scientific research can be easily conducted in the wild. Ex-situ populations kept at zoos provide opportunities to broaden research scenarios, benefiting conservation efforts^25–31^. African penguins breed well in captivity, and their stable ex-situ population could serve as a potential source for future reintroduction^21,30,32^.

To our knowledge, there is limited to no research on the breeding performance of captive African penguins that can be compared to the wild population, particularly studies conducted on a colony whose size reflects that of a wild population’s breeding grounds^25,26^.

Among over 60 institutions affiliated within European Association of Zoos and Aquaria (EAZA), colonies of African penguins typically consist of an average of 20–50 individuals. Interestingly, in the wild, colony sizes smaller than 50 individuals are observed to be the most prone to extinction according to the latest findings^8^. There are only 3 facilities that hold more than 100 penguins, with Zoo Wrocław being one of those few^15,32,33^. Since wild African penguins typically breed in colonies consisting of at least a couple of hundreds to thousands of individuals^21^, data from a large ex-situ colony may provide more accurate insights into breeding performance. This is because it creates conditions more similar in size and nesting space availability to those found in the wild breeding grounds.

Keeping and breeding animals at a zoo also has different limitations. European African penguin population is managed by the European Endangered Species Program (EEP) supported by EAZA. The EEP coordinator is responsible for managing the selection of mating pairs and making decisions on whether they should breed. In making these decisions, certain facts are essential. Some genetic lines may be overrepresented in population, some birds may be related, and some birds could have unknown heritage^15,16,32^. The later factor is of utmost importance, as it may mean that some penguins can carry a hybrid gene. Due to a historical fact of a cross-breeding between two different species—African Penguin *Spheniscus demersus* and Humboldt Penguin *Spheniscus humboldti*—and a lack of precise pedigree data for all individuals, only birds with a 100% known and hybrid-free pedigree can be bred in EAZA institutions^15,16,27,32^.

The extensive research on wild African penguins provides abundant information on their breeding parameters^18,23,34^, but it lacks a focus on intra-pair relations and their impact on these breeding parameters^16^. Among the various aspects of breeding behavior, the pair-bond duration factor is more easily observable and describable in ex situ conditions. African penguins are known to mate for life, and the majority of them do so^2,18,28,34,35^. However, there are documented cases of partner switching^26,27^.

The primary objective of this paper was to explore reproductive behavior in African penguins within a zoological setting. We scrutinized various breeding success parameters and drew comparisons with those observed in wild populations. Given the limited existing knowledge on the relation between the pair-bond and breeding success in African penguin in situ or ex situ colonies^16,36^, our research primarily focused on exploring interactions between those factors. Additionally, we investigated potential influences of changes in the colony’s size on these breeding parameters.

We hope that our findings can bring new insights for determining the breeding performance of the species and provide valuable information for population management and conservation efforts. To our knowledge, this is the first research that describes data collected over a long period of time (9 years) on the breeding of a large ex situ colony of over 100 individuals of African penguins *Spheniscus demersus* and the factors that could play a role in their breeding performance.

## Results

### Partner change

**Figure 1:**
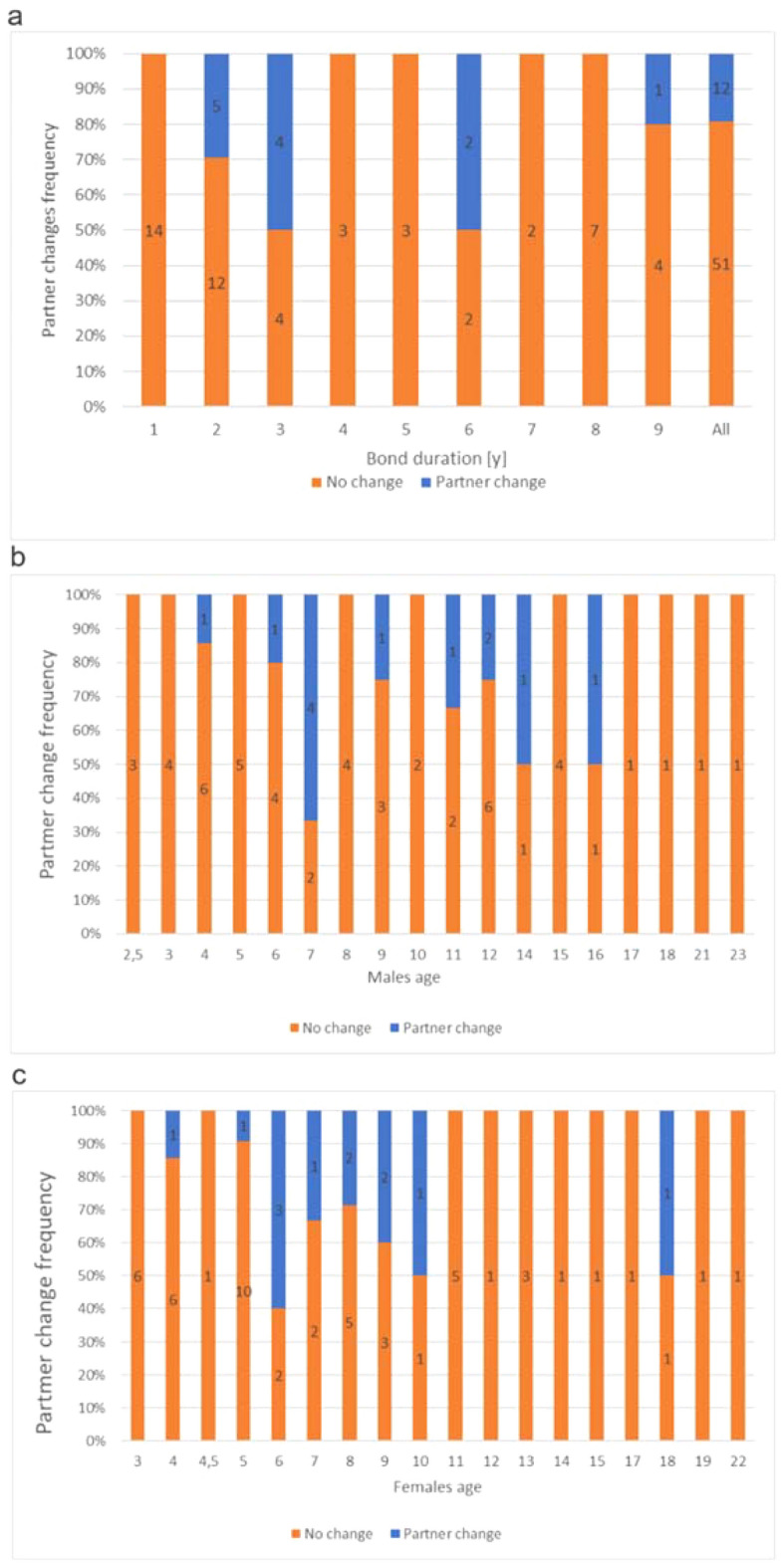
The percentage occurrence of a partner change in (**a**) all pairs in the colony and in pairs according to their bond duration in years; (**b**) males according to their age at the time of a partner change; (**c**) females according to their age at the time of partner change. Numbers on the columns indicate the number of individuals in each category.

The data on the occurrence of partner changes among pairs of penguins (Supplementary Table S1) indicate that this phenomenon happened in 19% of all pairs studied (12 out of 63 pairs, Fig. 1a). This observation aligns with the natural frequency of such situations in wild African penguins, where 80–90% of pairs typically remain with the same partner^2^. This alignment was statistically confirmed by a χ^2^ test (alpha = 5).

The logistic regression model examining the association between partner change and both bond duration in years and birds’ age at the time of change (male and female), yielded a non-significant result (OR 0.935, p = 0.64; OR 1.083, p = 0.34; OR 0.932, p = 0.43—respectively). Consequently, no further analyses (except GLM) were conducted. However, Fig. 1 illustrates the quantified data on a partner change in pairs with various bond duration and in male and female age at that time.

No partner changes were observed in pairs with bond durations of 1, 4, 5, 7 and 8 years. The highest frequency of changes occurred in pairs with bond durations of 2, 3 and 6 years (5 out of 17, 4 out of 8, 2 out of 4 pairs, respectively), with one occurrence in pairs with a bond duration of 9 years (1 out of 5 pairs). Males at the age of 2.5, 3, 10, 17, 18, 21, and 23 showed no partner changes. The highest frequency (4 out of 6) of changes occurred in males at 7 years old. In females, most partner changes happened between the ages of 6 and 10. Other age categories exhibited either no partner changes or singular occurrences.

The sample size was too small to run additional statistical analyses, but the following observations were noted (see Supplementary Table S1). In the 12 pairs that changed partners, 7 did so due to unsuccessful breeding, and 5 due to unknown factors; the latter are the case studies described in the next chapters. There were 21 cases in which one of the partners died or left the colony. These were not accounted for as “partner changes”, as the death or departure of a mate naturally necessitates a search for a new partner.

In 33 pairs with a breeding ban, and thus with no breeding success, 7 (21%) stayed together in spite of that fact. In these cases, the partner didn’t die or leave the colony. Two of these pairs had relatively short bond duration (1 and 2 years), while the other 5 (15%) pairs were bonded for as long as 5 to 9 years.

**Figure 2:**
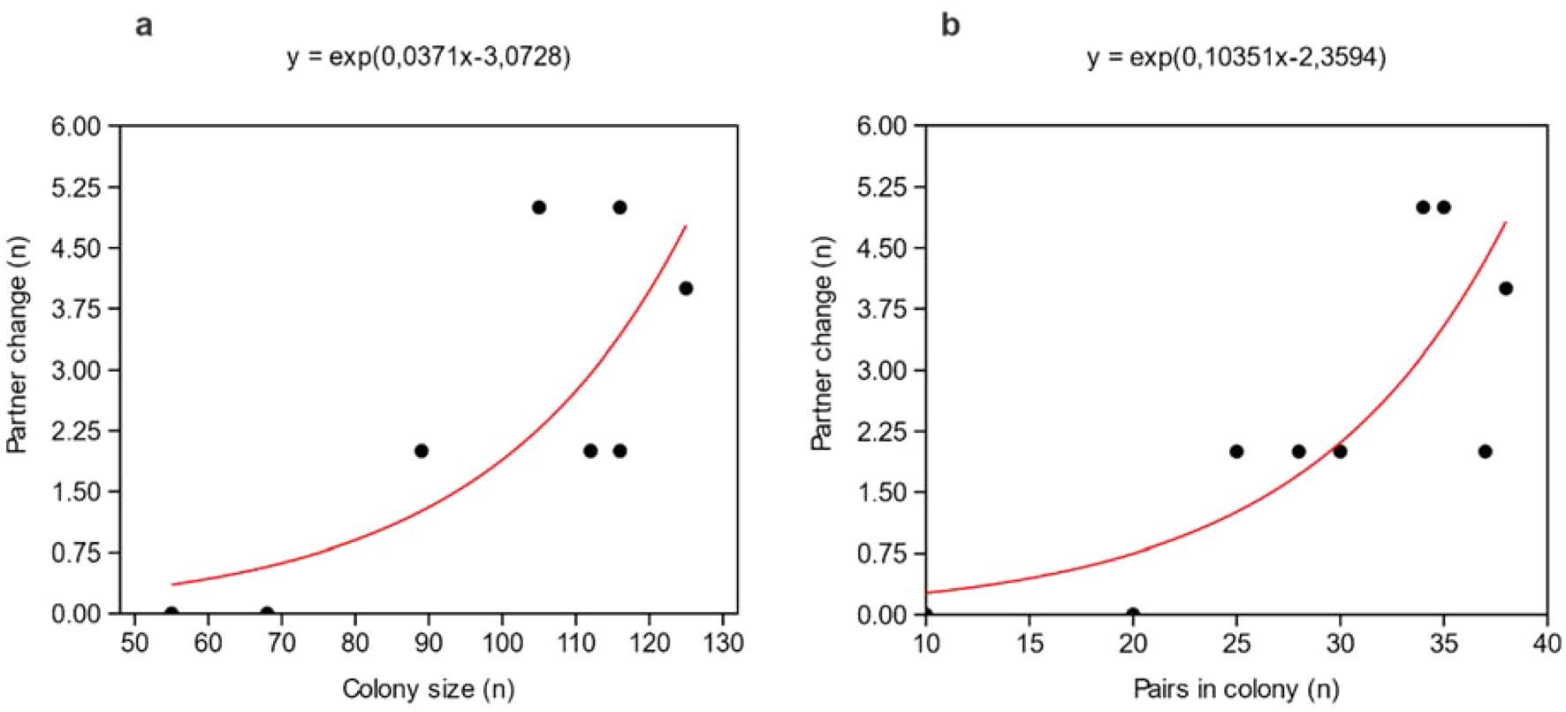
Relationship between the number of partner changes per breeding season in captive African penguins and (**a**) the size of the colony, and (**b**) the numbers of pairs in the colony. The red line shows the curve fitted to the relationship of the variables.

The GLM analyses (Fig. 2) showed significant relationship between the occurrence of a partner change and both increasing numbers of the colony and pairs in the colony (both p < 0.01).

### Breeding parameters

For the 30 breeding pairs of African penguins, reproductive parameters, including the numbers of eggs, chicks hatched and survived, as well as breeding success indicators such as clutch size, chicks hatched per breeding attempt, and the number of chicks fledged, along with their mean (± SD) are presented in Table 1. The latter were additionally supplemented with a confidence interval (95% CI). Additionally, brood size was calculated for breeding attempts with at least one hatchling and added to the table.

**Table 1:** Reproduction parameters (BA, eggs, CH, CS) and breeding success parameters (clutch size, CH per BA, chicks fledged, brood size) for breeding pairs of African penguins.

Pair	Male	M. age	Female	F. age	Bond	BA	Eggs	CH	CS	Clutch size	CH/BA	Chicks fledged	Brood size
1	Allen	2	Lilly	3	9	24	43	17	9	1.79	1.70	0.38	1.70
2	Pan Żółty	4	Pani Żółta	5	6	14	26	9	6	1.86	1.80	0.43	1.80
3	Roco	3	Coco	2	3	4	6	2	2	1.50	1.00	0.50	1.00
4	Sonny	5	Cher	9	9	21	43	24	21	2.05	2.00	1.00	2.00
5	Tucker	3	Ester	4	9	18	31	15	11	1.72	1.67	0.61	1.67
6	2Pac	13	Maya	10	8	28	53	9	5	1.89	1.50	0.18	1.50
7	Billy	4	Mandy	3	8	13	24	9	5	1.85	1.50	0.38	1.50
8	Brad	7	Angelina	3	8	16	28	8	3	1.75	1.14	0.19	1.14
9	Bruce	9	Pani Żółta	6	3	3	4	4	2	1.33	1.33	0.67	1.33
10	Jack Sparrow	7	Kapitan	14	8	18	29	3	2	1.61	1.00	0.11	1.00
11	Delfin	13	Pani Żółta	7	3	4	8	4	2	2.00	2.00	0.50	2.00
12	Hachico	5	Laura	6	7	15	30	12	9	2.00	1.71	0.60	1.71
13	Colin	3	Zyzia	2	6	13	24	6	5	1.85	1.50	0.38	1.50
14	Irek	5	Pani Żółta	7	2	2	4	2	2	2.00	2.00	1.00	2.00
15	Butthead	2	Szogunka	2	2	3	5	0	0	1.67	0.00	0.00	–
16	Hyzio	4	Nicki Minaj	3	2	9	14	6	1	1.56	1.50	0.11	1.50
17	Beavis	4	Szogunka	3	3	8	14	0	0	1.75	0.00	0.00	–
18	Butthead	4	Achad	3	2	3	6	0	0	2.00	0.00	0.00	–
19	Gofer	6	Jorgia	6	3	7	13	4	2	1.86	1.33	0.29	1.33
20	Rio	1.5	Terra	2	1	3	5	0	0	1.67	0.00	0.00	–
21	Roco	8	Mała Mi	3	3	9	17	8	7	1.89	1.60	0.78	1.60
22	Sonny	5	Sheloba	3	2	2	3	2	2	1.50	2.00	1.00	2.00
23	Stereo	3	Monica	7	1	2	4	2	1	2.00	2.00	0.50	2.00
24	Chowder	3	Barbossa	8	2	4	8	4	3	2.00	2.00	0.75	2.00
25	Hachico	5	Sheloba	4	2	2	5	0	0	2.50	0.00	0.00	–
26	Stereo	4	Harper	5	2	4	8	0	0	2.00	0.00	0.00	–
27	Batman	2	Henia	2	1	2	3	0	0	1.50	0.00	0.00	–
28	Bruce	17	Raya	6	1	2	3	0	0	1.50	0.00	0.00	–
29	Rainbow	1.5	Wusia	2	1	2	4	0	0	2.00	0.00	0.00	–
30	Rambo	2	Roli	2	1	3	6	0	0	2.00	0.00	0.00	–
**Mean**		5.1		4.7	3.93	8.60	15.70	5.00	3.33	1.82	1.08	0.35	1.61
** ± SD**		± 3.7		± 2.9	± 2.9	± 7.5	± 14	± 5.9	± 4.5	± 0.23	± 0.82	± 0.35	± 0.34
									95% CI	(1.74; 1.91)	(0.79; 1.36)	(0.23; 0.47)	(1.47; 1.75)

*M. Age/F. Age* male’s/female’s age at first breeding attempt at Zoo Wrocław, *Bond* pair bond duration in years, *BA* number of breeding attempts per pair, *Eggs* number of eggs laid per pair, *CH* number of chicks hatched per pair, *CS* number of chicks survived at least 3 months per pair, *Clutch size* an average number of eggs laid per pair per BA, *CH per BA* an average number of chicks hatched per pair per BA, *Chicks fledged* an average number of chicks fledged per pair per BA, *Brood size* an average number of chicks hatched per pair per a successful BA.

The age at first breeding attempt at Zoo Wrocław ranged from 1.5 to 17 years for males, and from 2 to 14 years for females. The analyzed bond duration varied from 1 to 9 years.

Spearman’s rank test (Table 2) revealed significant and strong positive correlations between bond duration and the number of breeding attempts, laid eggs, chicks hatched, and chicks survived. Mean clutch size exhibited no correlation with bond duration. CH per BA, the number of chicks fledged, and brood size all demonstrated a significant and moderately positive correlation with bond duration.

**Table 2:** Spearman’s rank correlation matrix of bond duration and numbers of breeding attempts (BA), laid eggs, chicks hatched (CH), chicks survived (CS), and means of clutch size, CH per BA, chicks fledged, and brood size in African penguin breeding pairs.

	Bond	BA	Eggs	CH	CS	Clutch size	CH/BA	Fledged
BA	0.90**							
Eggs	0.87**	0.98**						
CH	0.84**	0.81**	0.79**					
CS	0.83**	0.75**	0.74**	0.96**				
Clutch size	− 0.02	0.01	0.19	0.06	0.09			
CH/BA	0.40*	0.23	0.20	0.67**	0.70**	0.08		
Fledged	0.44*	0.25	0.22	0.66**	0.76**	0.07	0.94**	
Brood size	0.41*	0.31	0.31	0.71**	0.75**	0.23	0.91**	0.89**

Correlation coefficients marked with * and ** were statistically significant (*p < 0.05; **p < 0.01).

As the data on age at first breeding were not known for all individuals, this factor was not considered in correlation analyses. However, for birds that hatched at Zoo Wrocław and attempted breeding, observations were made and are discussed in later chapters.

### Similarities in breeding pairs

**Figure 3:**
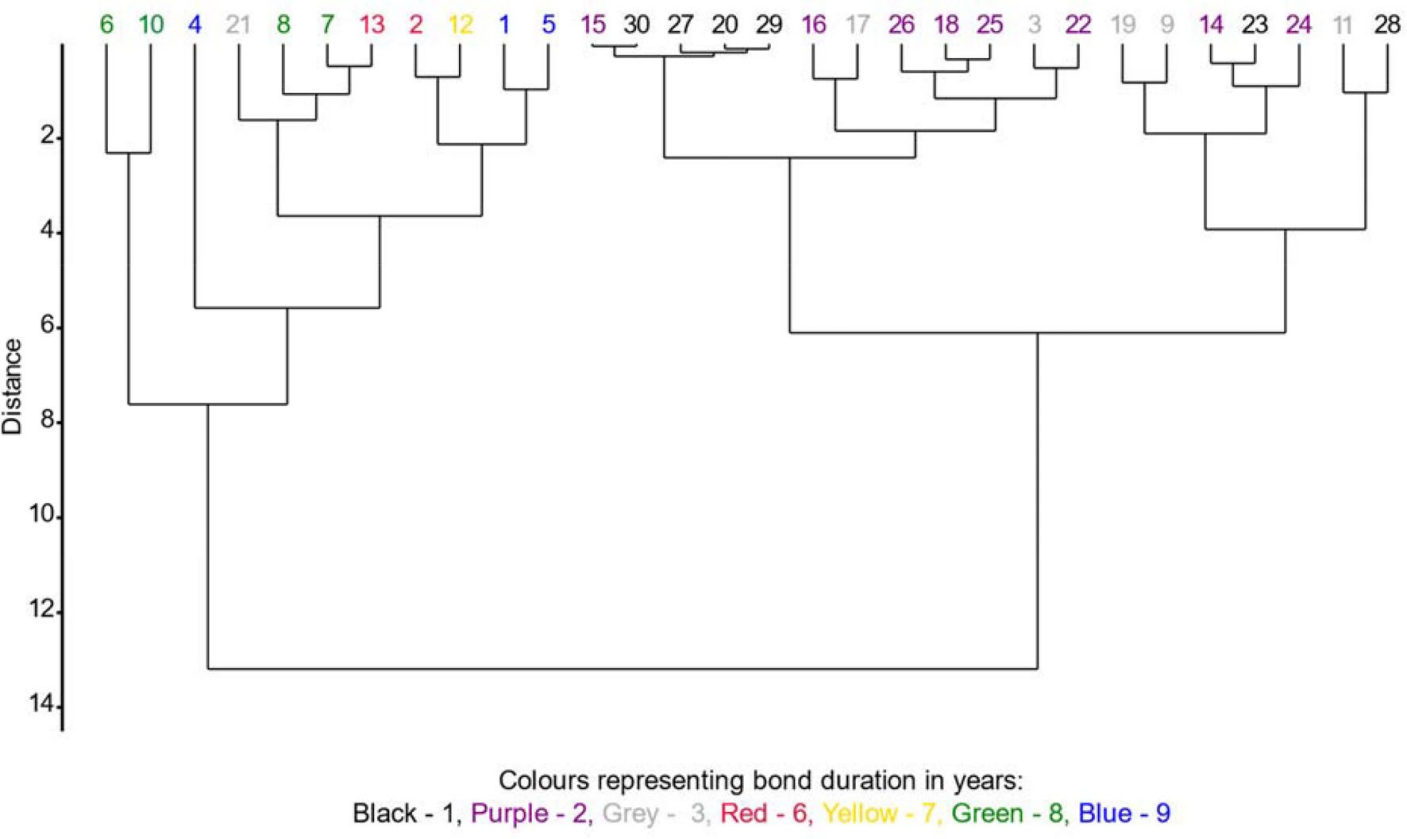
Dendrogram of similarities among breeding pairs of African penguins with the same bond duration in relation to all other breeding parameters. Numbers represent each pair from Table 1, colors represent each bond duration in years. The shorter the distance between pairs, the bigger the similarity among them.

The results of the clustering method are illustrated in Fig. 3, which depicts a dendrogram showing similarities among breeding pairs of penguins (from Table 1) with the same bond duration in relation to all other breeding parameters. Each pair is represented by a number on a dendrogram, and each color represents a different bond duration.

The multivariate analysis measured distances between pairs, with shorter distances indicating greater similarities in their breeding parameters. The pairs clustered into two distinct groups on the dendrogram: the right-hand side exclusively represented pairs with bond durations from 1 to 3 years, while the left-hand side represented pairs with bond durations from 6 to 9 years, with a single exception for pair 21 with a 3-year bond duration. In general, most pairs with similar bond durations exhibited close similarities in values of breeding parameters, although there were exceptions. Pairs 20, 29, 27, 30 with a 1-year bond were the most similar to each other and also to pair 15 with a 2-year bond. Among the 2-year bond pairs (18, 25, 26, 22, 16), similarities were observed, and they also showed resemblance to pairs 3 and 17 with a 3-year bond. Pairs 1 and 5, with the longest bond duration of 9 years, were similar to each other but differed significantly from pair 4 with the same bond duration. Pairs 6, 7, 8, and 10, all with an 8-year bond, formed two clusters, with pair 6 being similar to 10 and pair 7 being similar to 8, as well as pair 21 (3 years bond) and pair 13 (6 years bond). The lone pair with a 7-year bond, pair 12, showed similarity to pair 2 with a 6-year bond.

### Non-breeding pairs

The analysis of egg fertilization in non-breeding birds involved testing 177 eggs with 137 fertilized and 40 not fertilized. Males ages ranged from 2 to 25 years, and female ages from 2 to 22 years (Supplementary Fig. S1). T-Student tests indicated no statistically significant differences in egg fertilization based on either male or female age (t = 0.62, p = 0.54 for male age; t = 1.18, p = 0.24 for female age). Additionally, no statistically significant correlations were found among the same parameters (data not presented).

### Colony size

**Figure 4:**
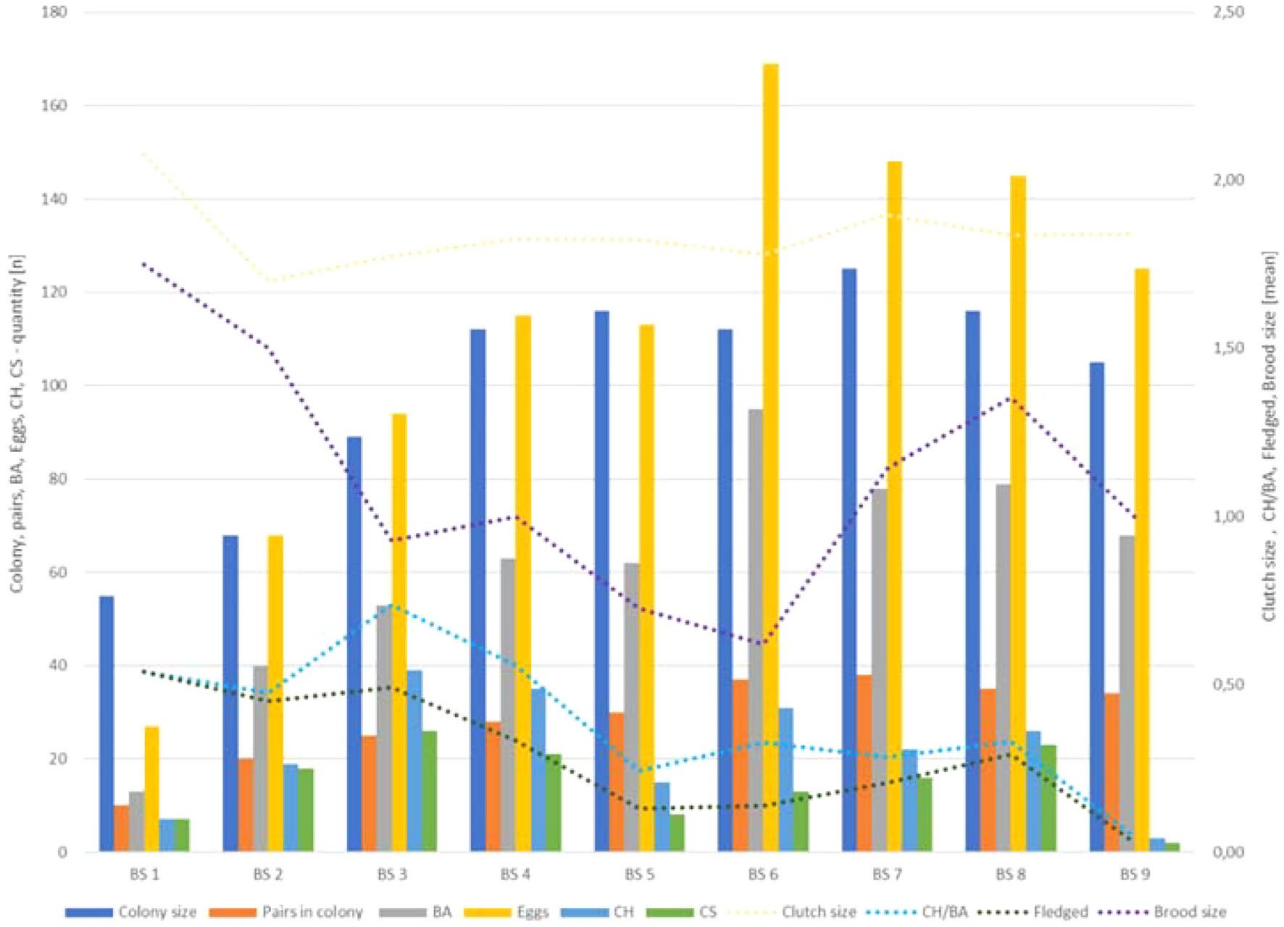
African penguin captive colony’s representations in numbers: Individuals in colony (colony size), pairs in colony, breeding attempts (BA), laid eggs, chicks that hatched (CH), chicks that survived (CS); and trends of mean values for clutch size, CH per BA, chicks fledged and brood size in each of analyzed breeding seasons (BS1–9).

Significant differences were found between the analyzed breeding seasons (χ^2^—116, p < 0.01, df = 40). Figure 4 illustrates the variations across seasons, where BS1 revealed the smallest numbers in colony size (55), pairs in the colony (10), breeding attempts (13), and egg count (27). The lowest number of chicks hatched (3) and survived (2) was observed in BS9. In contrast, BS7 showed the highest numbers in pairs (38) and colony size (125), while the highest numbers for breeding attempts (95) and eggs (169) were recorded in BS6. The numbers for chicks hatched (39) and survived (26) were highest in BS3. The same figure illustrates the trends of mean values for clutch size, chicks hatched per breeding attempt, chicks fledged, and brood size over the nine breeding seasons.

The Spearman’s rank correlation analysis (Table 3) revealed very strong, positive correlations among colony size, the number of pairs in the colony, the number of breeding attempts, and the number of eggs. Additionally, there were very strong correlations between the number of chicks hatched and the number of chicks survived, as well as between CH/BA and fledged chicks. A strong negative correlation was observed between the number of pairs in the colony and the mean number of chicks fledged. None of the other correlations were statistically significant.

**Table 3:** Spearman’s rank correlation matrix of colony size and number of pairs in colony between the numbers of breeding attempts (BA), laid eggs, chicks hatched (CH) and chicks survived (CS), clutch size, CH per BA, chicks fledged, and brood size.

	Colony size	Pairs in colony	BA	Eggs	CH	CS	Clutch size	CH/BA	Fledged
Pairs in colony	0.84**								
BA	0.72*	0.93**							
Eggs	0.75*	0.97**	0.98**						
CH	0.18	0.13	0.27	0.25					
CS	0.13	− 0.03	0.07	0.02	0.85**				
Clutch size	0.22	0.21	0.12	0.14	− 0.44	− 0.43			
CH/BA	− 0.51	− 0.61	− 0.46	− 0.49	0.64	0.64	− 0.28		
Fledged	− 0.61	− 0.72*	− 0.65	− 0.67	0.32	0.47	− 0.05	0.89**	
Brood size	− 0.36	− 0.42	− 0.44	− 0.46	− 0.38	0.00	0.44	0.19	0.56

Correlation coefficients marked with * and ** were statistically significant (*p < 0.05; **p < 0.01).

## Discussion

Our study is, to the best of our knowledge, the first to report the influence of pair bond duration and colony size on breeding success in captive African penguins (*Spheniscus demersus*) housed in a colony that mirrors conditions comparable in size and nest site availability to those found in their natural habitat.

### Partner change

As with all penguin species, African penguins are generally recognized for forming lifelong and monogamous bonds^16^. However, in up to 20% of wild pairs^2,18^, partner changes may occur, primarily prompted by unsuccessful breeding attempts with no shortage of nesting sites^2^. Wild penguins establish their pair bond annually upon returning to the breeding grounds. This pattern is attributed to their behavior outside the breeding season when they spend time in large groups foraging in the open ocean, making the maintenance of bonding behaviors challenging and unnecessary^2,28^. The captive African penguin population at Zoo Wrocław exhibited a comparable frequency of partner changes, despite the birds remaining in their breeding territory throughout the year.

According to the logistic regression model, the number of partner changes showed no relation to pair bond duration in years or birds’ age in our captive colony. This prompts further investigation into data that was too small for in-depth analyses but provided interesting observations.

Only 7 out of 12 partner changes were attributed to unsuccessful breeding, believed to be the main reason for such changes in the wild^2^. The other 5 pairs presented intriguing case studies where, despite successful breeding, individuals still decided to change partners (see Supplementary Table S1).

In the initial case, a female (5 year old), Pani Żółta (“Miss Yellow”), formed a pair with Pan Żółty (“Mr. Yellow”) (4 y.o.). They had four unsuccessful breeding attempts, leading to a three-year separation, after which they reunited. During the separation, Mr. Yellow was paired with a female with a breeding ban, resulting in no breeding success. This lack of success may have prompted his return to his former partner for another breeding attempt. Meanwhile, Miss Yellow was paired with three different males during the separation, successfully raising chicks with each. After an unsuccessful attempt with Mr. Yellow in BS2, she raised chicks with Bruce. In early BS3, she repeated breeding success with Bruce and changed to Delfin to successfully raise chicks later in the same season. In early BS4, she successfully bred with Irek and later raised chicks with Bruce again. In BS5, she started anew with Irek, and later repeating successful breeding with Delfin. Finally, in BS6, she reunited with Mr. Yellow and has bred successfully solely with him since then. Breeding with different males occurred in their own separate nests located in close proximity. Remarkably, no aggressive behavior among these males was observed, and the involved parties regularly visited each other throughout the years.

In a different pair, Sonny and Cher, with an 8-year-long pair bond, we observed the male changing partners and successfully breeding with a very young, 3-year-old, female named Sheloba in late BS8, after raising chicks with Cher earlier in the season. Interestingly, in the previous season, Sheloba was observed in the same nest with Sonny, likely attempting to breed. After a successful BS8, Sheloba changed to Hatchicko but had no success breeding with him.

Those cases align with the general theory of mate selection, where females are more frequently the determinants. This alignment corresponds to their larger investment in offspring, characterized by a limited gamete production capacity per breeding period. In contrast, males can produce millions of gametes, allowing them to fertilize multiple females. This asymmetry underscores the significant role of females in the mate choice process^16^.

As we observed, a partner change could serve as a solution for unsuccessful breeding, and it might also enhance breeding success for females, particularly when the food supply is secure.

Certainly, more research is required to observe intra- and extra-pair relations in wild African penguins, challenging the perception of their presumed monogamy. This prompts the question of whether African penguins, despite their initial monogamous tendencies, are prone to adopting alternative breeding strategies, such as polyandry, under favorable conditions.

Most research on wild African penguins primarily emphasizes their monogamy without exploring other intra- or extra-pair relations^2,5,20,34^. Furthermore, the research focusing specifically on pair bond duration in wild African penguins is scarce^16,36^. Galante and Margulis^29^ describe pair bonds in captive Humboldt penguins, primarily focusing on behaviors indicating pair-bond strength. In captive African penguins, Modesto et al.^27^ provide some insight into partner changes as they explore extra-pair mating in their genetic studies, while Baciadonna et al.^28^ describe pair bonds in the context of vocal partner recognition. None of these captive-based studies explore the relationship between pair bonds and breeding success.

To our knowledge, no specific research focuses on this topic, making our findings preliminary observations for future research, which may be crucial for both captive population management and conservation efforts.

The logistic regression model showed no association between the number of partner changes and birds age at our captive colony. Since, to our knowledge, there is no data describing this relation in the wild African penguins, no further analyses were attempted.

It is worth mentioning that our study revealed an increasing occurrence of partner changes with a higher number of individuals and pairs in the colony (Fig. 2). This suggests that in a small colony, penguins may pair up with available partners for breeding attempts. However, as the colony size grows and more potential partners become available, penguins might be inclined to change partners in order to find a more suitable mate or to enhance their breeding success by attempting breeding with more than one partner.

Since African penguins are generally believed to seek another partner after losing a mate^36^ or experiencing unsuccessful breeding^2^, it is crucial for these individuals to have the opportunity to choose a new mate. As Fig. 2 demonstrates, an increasing colony size will provide them with the chance to find a new partner, hence regain their breeding success. Given that diminishing food supplies are one of the main causes for the current decline in wild colonies^8,18^, this information might be crucial for conservation strategies. The lack of an opportunity to choose a new breeding mate and to restore breeding success might be one of the factors responsible for the increasing extinction rate in smaller colonies^8^.

Similar concern may arise from our observation of pairs that did not change a partner in spite the unsuccessful breeding. 15% of those pairs maintained extensive pair bonds lasting 5 to 9 years, without attempting any partner changes. This resilience in the pair bond, preventing divorce even without breeding success, could potentially make wild penguin populations more susceptible to extinction.

Modesto et al.^27^, in their study on the molecular genetics of African penguins, discovered that unnoticed extra-pair mating could be a significant factor in revealing unknown family relationships in a captive colony. Given our observations, genetic analyses may prove crucial for captive population management. Such analyses could confirm pedigrees and ascertain whether penguins with a breeding ban have mated outside their pair, potentially resulting in unfavorable offspring (such as hybrids or inbred individuals). Additionally, future research could explore whether extra-pair mating in captive populations may function as a mechanism to restore breeding success for individuals while maintaining the bond with the original partner with a breeding ban.

### Breeding parameters

Clutch size, representing the number of eggs laid in a single breeding attempt, serves as a crucial indicator of a species’ reproductive behavior. In wild African penguins, this parameter ranges from 1.71 to 1.92, with mean values 1.82^34^, 1.84^23^ or 1.86^18^. Our captive colony’s mean clutch size of 1.82 aligns closely with these values.

Another parameter describing breeding success is the number of chicks hatched per nest, ranging from 0.86 to 1.04 across Namibian localities, with an overall mean of 0.95^23^. Comparing this to our calculations for chicks hatched per breeding attempt (1.08), the data from Namibian localities falls within our confidence interval (0.79–1.36), suggesting no significant disparities.

The mean number of chicks fledged per breeding attempt is a prevalent indicator of breeding success in wild African penguins. In Namibia, this parameter varies from 0.15 to 0.73, with an overall mean of 0.61^23^, while Robben Island, South Africa, shows a range of 0.32 to 0.59 and an overall mean of 0.47^18^. Our captive colony exhibits a similar parameter with a mean value of 0.35, aligning with data from Robben Island.

Exploring the less common parameter of brood size, calculated as the number of downy chicks per nest^34^, we found that our captive colony’s mean brood size (1.61) mirrored the findings from Robben Island during a recolonization period (1.52 to 1.71)^34^.

Our research focuses on the breeding parameters of the captive colony, utilizing comparisons to wild populations for contextual understanding. Clutch size, chicks hatched per breeding attempt, fledged chicks, and brood size are pivotal parameters assessed in both settings. Notably, the information from the wild consistently falls within the confidence intervals derived from our captive bird research (see bottom of Table 1), affirming the comparable reproductive parameters between the two populations. This underscores the significance of studying captive populations in shaping effective conservation strategies and management practices for this species.

The information on potential previous breeding for birds that arrived at Zoo Wrocław as adults was lacking, preventing a comprehensive analysis of the age of first breeding in our captive colony and comparison with the wild population, where it averages at around 4 years old^37,38^. However, observations from birds hatched at Zoo Wrocław revealed that captive individuals as young as 1.5 years old attempted breeding. This aligns with Crawford’s et al.^18^ findings on Robben Island, where fish availability was abundant at that time. He described a remarkably young bird (1 year and 8 months old) attempting to breed but not maintaining the task, ultimately abandoning the nest after about 2 weeks.

Studies on wild seabirds indicate that young birds take considerable time before investing in breeding. They prioritize acquiring survival skills, particularly perfecting foraging abilities, to prepare for energy-costly breeding attempts^39–41^. Since acquiring sufficient amounts of food at a captive colony is not a struggle, it may suggest that securing sufficient feeding grounds in the wild can benefit the declining wild population by encouraging birds to attempt breeding at younger age.

Our analysis of additional data from eggs laid by captive African penguin pairs with a breeding ban showed no statistically significant influence of age on the probability of egg fertilization. The age of birds producing fertilized eggs ranged from 2 to 25 years old. With no similar studies for comparison with captive or wild populations, it can only be assumed that captive African penguins can produce a fertilized egg at a very young age and throughout most of their lives. However, the mere occurrence of egg fertilization doesn’t guarantee breeding success, as none of the breeding pairs in our colony, where both partners were 2 or younger, had any success in hatching chicks. Success improved in pairs where at least one partner was older than 2, aligning with the review suggesting that for birds breeding for the first time, their success could improve if they pair up with a more experienced partner^41^.

As Fowler^42^ mentions, many long-lived birds that have a clear relationship between age and breeding success form long term pair bonds which are positively correlated to breeding success. He presents the pair bond investment hypothesis, asserting that maintaining a long-term relationship over multiple breeding seasons correlates with higher breeding success parameters. The benefits of a long-term pair bond include shared experience, more equally distributed parental investment, and lower energy consumption. Bonded birds are more efficient in nest-building, clutch care, and brood rearing, requiring less energy before the breeding season for courtship. The hypothesis is further supported by increased energy costs in the case of a divorce or the death of a partner, leading to a decline in breeding success^42^.

Our findings support the mentioned theory, as the analysis for all breeding pairs revealed strong and significant positive correlation between pair bond duration and all reproductive parameters (numbers of BAs, eggs, chicks hatched and chicks survived). While the correlation between pair bond duration and reproductive parameters may seem obvious (as those parameters naturally cumulate over time), it is essential to emphasize its significance in light of the lack of prior data on this matter in published literature. Moreover, the most significant finding emerges from correlation analyses with indicators of breeding success. We identified a positive correlation between the number of chicks hatched per breeding attempt, chicks fledged, brood size, and the pair bond duration. Clutch size revealed no correlation with bond duration, highlighting its strict relation to species biology rather than pair-bond duration. Our multivariate analysis for breeding pairs consistently presented similar findings. Notably, when comparing pairs with similar breeding parameter values, it is evident that these pairs also share similar pair-bond durations. Pairs with short bond durations (1–3 years) significantly differ from those with long pair-bond durations (4–9 years), indicating differences in their breeding parameter values (Fig. 3).

Our research contradicts the notion that there is little evidence for improvement in breeding success with an increased pair bond duration in penguins, as suggested by EAZA’s *Spheniscus* penguin husbandry manual^16^. We found that a longer pair bond duration is a significant factor for sustaining breeding success and, consequently, population numbers in African penguins.

The earlier mentioned pair bond investment hypothesis^42^ suggests that African penguin may be vulnerable to abrupt changes in population dynamics. Any sudden environmental changes, such as disease outbreaks, environmental disasters, predation, or other catastrophic events that result in adult mortality and pair breakage, would contribute to a decline in population numbers^8,18,20^. This decline would not only be due to the loss of one individual but also result from increased costs for the remaining partner in the search for a new mate, leading to a reduction in their breeding success^20,42^. Furthermore, the decrease in population numbers diminishes the likelihood of finding a new partner, thereby accelerating the pace of population decline. These unveiled observations from captive African penguins might provide additional reasoning for promoting enhanced protection of their wild environment against future catastrophic events.

### Colony size

Analyzing the growth in colony size in captive conditions presents challenges, primarily dictated by factors such as enclosure size and its predetermined maximum capacity, subject to specific regulations set by each EAZA facility housing African penguins^16^. Compliance with recommendations from the EEP coordinator further influences colony size management^32^. The penguin colony at Zoo Wrocław, designed for 100–120 individuals, exhibited notable growth, doubling in size from the founding group of 55 penguins to 112 individuals in BS4 within a span of four years, hovering near its capacity thereafter, with a peak of 125 penguins in BS7. Animal husbandry techniques, including dummy egg swapping and relocating surplus individuals, were implemented to manage the colony as it reached its capacity. Consequently, post-capacity fluctuations in colony size could not be analytically compared to wild records. However, the initial four years of growth allowed for a comparison to the recolonization of Robben Island by African penguins, where the population surged from 9 pairs in 1983 to 227 in 1986. The rapid growth at a new colony site was attributed to the abundance of sardine and anchovy stocks^34,43,44^. Noteworthy similarities emerged in both colonies regarding the correlation between increasing numbers of individuals and pairs in the colony (r = 0.84 at Zoo Wrocław; r = 0.94 at Roben Island^18^).

The size of the colony and the number of pairs at Zoo Wrocław demonstrated strong positive correlations with numbers of breeding attempts and eggs laid. These initial breeding parameters were chosen to describe overall reproductive performance, as husbandry techniques prevented consideration of other breeding parameters, and the mean clutch size showed no significant correlations. Barbosa et al.^45^ found that larger sub colonies of Chinstrap penguins exhibited better breeding success than smaller ones. Sherley et al.^43^ noted in the wild that a larger colony size offers more protection from predation, and aggression between neighboring penguins becomes a factor only after surpassing maximum capacity. Nest form is crucial in the wild; bush or open nests are harder to defend, while burrow nests are easier as only the entrance needs protection^43^. In Zoo Wrocław, with no predator concerns and nest availability adjusted with the rising colony and pairs, the provided igloo-like nests allowed penguins to easily protect themselves from neighbors.

Given that penguins at Zoo Wrocław were fed ad-libitum throughout the studied years and additional nests were provided as needed, it can be assumed that the captive colony experienced optimal conditions for rapid population growth. This suggests that, in general, African penguins have the potential for rapid population increase when provided with sufficient food and nesting grounds. Considering that the lack of nesting opportunities withing colonies is not a concern for wild African penguins^8,18,23,34^, our findings could be of significant importance for species conservation efforts. They suggest that securing suitable fishing grounds for wild penguins is crucial for population recovery, particularly given the significance of colony size in breeding success, as indicated by recent research^8^. The population decline not only reduces colony sizes but also leads to fragmentation into smaller units. Smaller colonies face a higher risk of extinction, as highlighted by Crawford’s et al.^8^ research, indicating that a colony size below 250 individuals has an almost 100% probability of extinction in the next 40 years. Conversely, a colony with over 5000 individuals would have a 100% chance of survival in that time frame. Unfortunately, none of the existing wild populations currently reach that number, and the seven largest colonies, with populations between 1000 and 5000 individuals, have a 67% chance of extinction by 2059^8^.

## Conclusions

Captive African penguins typically display monogamous behavior, attempting to breed with available partners. Strong pair fidelity may persist even after unsuccessful breeding attempts. Alternatively, they may change partners if a more suitable one becomes available and engage in extra-pair mating to enhance breeding success while maintaining the original bond. That underscores the necessity for more in-depth studies of both intra and extra-pair relations for a comprehensive understanding of colony-wide breeding success. Genetic analyses are essential for uncovering unknown family relations and confirming pedigrees, particularly for captive populations considered for future wild population restoration.

Provided sufficient food African penguins may initiate breeding at 1.5 years old. They can produce fertilized eggs at 2 years old, but breeding success requires experience.

The duration of pair bonds emerges as crucial for enhancing the reproductive success of captive African penguins and arises as significant factor for conservation strategies. Incidents such as oil spills, causing sudden increases in adult mortality, result in pair breakage and subsequent population decline. This challenges remaining partners in securing new mates, thereby reducing breeding success and accelerating overall population decrease. Thus, protecting the natural environment from sudden catastrophic events is essential for species conservation.

Captive African penguins exhibit rapid population growth with ad-libitum food and proper housing, emphasizing the need to secure suitable fishing grounds for wild population recovery, particularly given the recent acceleration of the extinction rate.

Our study on a large captive colony of African penguins reveals significant similarities to their wild counterparts, emphasizing the importance of maintaining captive populations that closely mirror natural conditions for the welfare of the birds.

## Materials and methods

### Animals and research site

The colony of African penguins *Spheniscus demersus* has been housed at Zoo Wrocław in Poland since 2014. The exhibit, occupied by the birds, comprises a 900 m^2^ beach area and nearly 1000 m^2^ pool surface. The pool, with a depth of 4.5 m, holds a volume of 2460 m^3^ of water. Originally, the colony consisted of 55 individuals brought into the zoo in 2014 from 4 different institutions: Zoo Gdańsk, Zoo Banham, Zoo Bristol, and Zoo Pont-Scorff. At the time of writing this article, the colony has expanded to include 99 penguins. The zoo lacks specific information regarding the breeding history or initial pairing status of the penguins that were introduced in 2014.

### Data collection

The data considered in this study spans nine consecutive breeding seasons from 2014 to 2023. Although African penguins may breed throughout the entire year, in European zoos, they typically experience a peak in the breeding season that overlaps two calendar years. This peak begins in early fall (as soon as August) and extends until late spring (May)^16^. As a result, each breeding season was characterized as:

1. BS1—Breeding season 1 (2014–2015).
2. BS2—Breeding season 2 (2015–2016).
3. BS3—Breeding season 3 (2016–2017).
4. BS4—Breeding season 4 (2017–2018).
5. BS5—Breeding season 5 (2018–2019).
6. BS6—Breeding season 6 (2019–2020).
7. BS7—Breeding season 7 (2020–2021).
8. BS8—Breeding season 8 (2021–2022).
9. BS9—Breeding season 9 (2022–2023).

A total of 191 birds were considered over the years 2014–2023, comprising 91 males, 97 females, and 3 birds of unknown sex, with ages ranging from 1 month to 22 years.

All the birds analyzed for this study had known age and sex, obtained from the Zoo Wroclaw’s and ZIMS—Species 360 databases^33^.

Among them, 55 individuals were colony founders, and 137 hatched at Zoo Wrocław. The data on egg laying, hatching or swapping for dummy eggs were meticulously collected throughout the years 2014–2023 by the keepers. All the nests were checked daily and the changes recorded.

For pair formation, we considered two birds that have attempted on building a nest and have laid at least one egg.

Supplementary Table S1 presents the zoo’s 9-year record of all penguin pairs’ breeding attempts, distinguishing between pairs allowed to breed (highlighted in bold) and those with a breeding ban (normal font)^32^. Some pairs remained constant, while others experienced partner changes due to reasons such as mate death, leaving the colony, unsuccessful breeding, and unknown factors.

All penguin pairs that were prohibited from breeding had their eggs removed and replaced with dummy eggs. For the last 3 breeding seasons (BS7–BS9), the removed eggs were examined to determine if they were fertilized. Evaluation of the fertilization was based on identification of the presence of the embryo, vascular field or assessment of the macroscopic appearance of the blastodisc. Fertilized eggs typically exhibit large, round blastodiscs with clearly visible *area pelicula* and *area opaca*, while unfertilized blastodiscs are smaller, asymmetrical, sometimes with vacuoles^46,47^.

The changing size of the colony, along with the varying numbers of pairs, their breeding attempts and, overall breeding performance for each year (breeding season) is presented in Supplementary Table S2. The breeding performance is characterized by the breeding parameters: following factors (measures of breeding performance): the number of eggs laid by the pair, the number of chicks that hatched from those eggs and the number of chicks that survived at least 3 months, and breeding success indicators—clutch size, chicks hatched per breeding attempt, chicks fledged per breeding attempt and brood size.

### Statistical analysis

#### Partner change

To assess the strength of pair bonds within the colony, the data from Supplementary Table S1 were analyzed for the frequency of a partner change occurrence using χ^2^ test. The results were than compared with the natural frequency, suggesting that 80 to 90% of African penguins mate for life^2^. Our research aimed to evaluate the partner change in the context of pair fidelity. Therefore, the partner change was only considered for birds that underwent partner changes due to reasons other than the partner dying or leaving the colony. The logistic regression model was applied to explore the relationship between partner change and pair bond duration as well as birds’ age at that time. Birds’ age was calculated as a sum of an individual’s age at first breeding with particular partner and the duration of pair’s bond.

Observations of a partner’s death or departure were also quantified. Reasons for a partner change were categorized as unsuccessful breeding or unknown. The latter instances served as case studies, as discussed in the following sections.

To assess if a partner change occurs more frequently with a changing colony size and varying number of pairs in the colony, we applied a Generalized Linear Model with Poison distribution and logarithmic link functions.

#### Breeding parameters

Because not all birds were permitted to breed, only the pairs highlighted in bold in Supplementary Table S1 were included in subsequent analyses (see Table 1). Data for breeding pairs were supplemented with reproduction parameters describing their breeding performance (numbers of: breeding attempts, eggs laid, chicks hatched and, chicks survived at least 3 months). These parameters were utilized to calculate breeding success indicators: clutch size, chicks hatched per breeding attempt, chicks fledged per breeding attempt in each pair, which were then analyzed for correlations with bond duration. Additionally, brood size was calculated for successful breeding attempts.

Shapiro–Wilk test (p < 0.05) was used for checking for normality of data distribution. Since the criteria was not met—a nonparametric Spearman’s rank test was used to check for correlations.

#### Similarities in breeding pairs

The clustering method was employed to validate that bond duration influences breeding performance parameters, given that pairs with similar values of breeding parameters also exhibit the same or similar bond duration. The data from Table 1 were utilized to identify similarities among pairs of birds with the same bond duration, considering all reproductive parameters as variables. Classical clustering Ward’s method was employed after data standardization.

#### Non-breeding pairs

For the non-breeding pairs enlisted in Supplementary Table S1, the assessment focused on the fertilization status of eggs laid during breeding seasons BS7–BS9. Following this evaluation, statistical analyses were undertaken to investigate the impact of age on egg fertilization. A T-Student test was run to see if there is a difference between fertilized and unfertilized eggs based on the age of birds. Subsequently, Spearman’s rank test was employed to identify any potential correlations between egg fertilization and the age of the birds.

Analyses were conducted separately for male and female age.

#### Colony size

The collective data for all breeding seasons from Supplementary Table S2 facilitated an investigation into potential correlations between the changing size of the colony and the varying number of pairs within the colony with the overall breeding performance. To scrutinize these dynamics, a χ^2^ test was employed to detect significant differences among breeding seasons. Additionally, a nonparametric Spearman’s rank test was conducted to identify correlations.

All the statistical analyses mentioned above were executed using the Past Statistical software^48^ and R^49^.

### Supplementary Information

Supplementary Information.

## Data Availability

The datasets used and analyzed during the current study are available from the corresponding author on reasonable request.
