# Supplementary material for: Breeding behavior analysis in a large captive colony of African penguins (Spheniscus demersus) and its implications for population management and conservation
Source: Sci Rep. 2024 Feb 13;14:3589. doi: 10.1038/s41598-024-54105-w (PMC10864276; doi:10.1038/s41598-024-54105-w)
Supplement: Supplementary file 1 — Supplementary Information. [file 41598_2024_54105_MOESM1_ESM.docx]

Table S1: Basic data on all pairs of African penguins at Zoo Wrocław.

| Pair no. | Male | M. Age | Female | F. Age | Bond (y) | 1^st^ BA | BAs (n) | Partner change | Change reason |
| --- | --- | --- | --- | --- | --- | --- | --- | --- | --- |
| **1** | **Allen** | **2** | **Lilly** | **3** | **9** | **BS1** | **22** | **no** |  |
| **2** | **Pan Żółty** | **4** | **Pani Żółta** | **5** | **6** | **BS1** | **13** | **BS2** | **Unsuccessful BA ^a^** |
| **3** | **Roco** | **3** | **Coco** | **2** | **3** | **BS1** | **4** | **BS4** | **Partner** **died** |
| **4** | **Sonny** | **5** | **Cher** | **9** | **9** | **BS1** | **17** | **BS8** | **Unknown ^b^** |
| **5** | **Tucker** | **3** | **Ester** | **4** | **9** | **BS1** | **17** | **no** |  |
| 6 | Sven | 3 | Emma | 4 | 9 | BS1 | 18 | no |  |
| 7 | Duku | 3 | Dwukropka | 3 | 2 | BS1 | 2 | BS3 | Partner died |
| 8 | Grześ | 6 | Linda | 5 | 9 | BS1 | 19 | no |  |
| 9 | Sasha | 6 | Anja | 2 | 6 | BS1 | 8 | BS6 | Partner died |
| 10 | Boss | 15 | Harpia | 7 | 8 | BS1 | 19 | BS9 | Partner died |
| 11 | Chandler | 2 | Monica | 12 | 5 | BS2 | 7 | BS6 | Partner died |
| **12** | **Jack Sparrow** | **7** | **Kapitan** | **14** | **8** | **BS2** | **18** | **no** |  |
| 13 | Pirat | 6 | Barbossa | 2 | 6 | BS2 | 9 | BS7 | Unsuccessful BA |
| 14 | Asier | 3 | Andrea | 4 | 5 | BS2 | 8 | BS6 | Partner died |
| 15 | Ross | 8 | Rachel | 11 | 8 | BS2 | 14 | BS8 | Partner died |
| **16** | **Brad** | **7** | **Angelina** | **3** | **8** | **BS2** | **16** | **no** |  |
| **17** | **2Pac** | **13** | **Maya** | **10** | **8** | **BS2** | **30** | **no** |  |
| **18** | **Bruce** | **9** | **Pani Żółta** | **6** | **3** | **BS2** | **3** | **BS3 BS4** | **Unknown ^c^** |
| **19** | **Billy** | **4** | **Mandy** | **3** | **8** | **BS2** | **14** | **no** |  |
| 20 | Robie | 4 | Paige | 3 | 8 | BS2 | 17 | no |  |
| 21 | Mamma | 3 | Jorgia | 3 | 2 | BS3 | 5 | BS5 | Partner died |
| 22 | Hachico | 5 | Carla Bruni | 7 | 4 | BS3 | 9 | BS6 | Partner left |
| **23** | **Hachico** | **5** | **Laura** | **6** | **7** | **BS3** | **15** | **no** |  |
| 24 | Muminek | 3 | Migotka | 3 | 7 | BS3 | 18 | no |  |
| 25 | James | 13 | Dwukropka | 4 | 1 | BS3 | 2 | BS4 | Partner died |
| **26** | **Delfin** | **13** | **Pani Żółta** | **7** | **3** | **BS3** | **4** | **BS3 BS5** | **Unknown ^c^** |
| 27 | Pan Żółty | 6 | Wampirka | 7 | 4 | BS3 | 8 | BS7 | Partner died |
| **28** | **Irek** | **5** | **Pani Żółta** | **7** | **2** | **BS4** | **2** | **BS4 BS5** | **Unknown ^c^** |
| **29** | **Colin** | **3** | **Zyzia** | **2** | **6** | **BS4** | **13** | **BS9** | Partner died |
| 30 | Pierre | 5 | Rebel | 1,5 | 3 | BS4 | 4 | BS7 | Partner left |
| **31** | **Butthead** | **2** | **Szogunka** | **2** | **2** | **BS5** | **3** | **BS6** | **Unsuccessful BA** |
| 32 | Hoyt | 3 | Harper | 2 | 4 | BS5 | 8 | BS8 | Partner left |
| 33 | Cucho | 2 | Janush | 3 | 1 | BS5 | 1 | BS5 | Partner died |
| 34 | Kamil | 3 | Charlie | 3 | 5 | BS5 | 12 | no |  |
| **35** | **Hyzio** | **4** | **Nicki Minaj** | **3** | **2** | **BS5** | **9** | **no** | **Both left** |
| **36** | **Butthead** | **4** | **Achad** | **3** | **2** | **BS6** | **3** | **no** | **Both left** |
| 37 | Casey | 3 | Alaska | 2 | 2 | BS6 | 4 | no | Both left |
| 38 | Ru Paul | 2 | Freya | 3 | 2 | BS6 | 6 | no | Both left |
| 39 | Loki | 3 | Gwen | 3 | 2 | BS6 | 6 | no | Both left |
| 40 | Chuck | 3 | Leeloo | 2 | 2 | BS6 | 4 | no | Both left |
| 41 | Suomi | 2 | Mono | 2 | 2 | BS6 | 2 | no | Both left |
| 42 | Alex | 3 | Rosie | 3 | 1 | BS6 | 1 | BS7 | Partner died |
| **43** | **Rio** | **1,5** | **Terra** | **2** | **1** | **BS6** | **3** | **no** | Both left |
| **44** | **Roco** | **8** | **Mała Mi** | **3** | **3** | **BS6** | **9** | **no** |  |
| 45 | Asier | 7 | Milagros | 2 | 1 | BS6 | 2 | BS7 | Partner left |
| **46** | **Beavis** | **4** | **Szogunka** | **3** | **3** | **BS6** | **8** | **BS7 BS8 BS9** | **Unsuccessful BA ^d^** |
| **47** | **Gofer** | **6** | **Jorgia** | **6** | **3** | **BS6** | **7** | **no** |  |
| **48** | **Sonny** | **5** | **Sheloba** | **3** | **2** | **BS7** | **2** | **BS8** | **Unknown ^e^** |
| 49 | Cucho | 6 | Szogunka | 5 | 3 | BS7 | 5 | BS7 BS8 BS9 | Unsuccessful BA ^d^ |
| 50 | Clyde | 3 | Kohana | 3 | 1 | BS7 | 3 | BS8 | Partner left |
| 51 | Ross | 16 | Kohana | 4 | 1 | BS7 | 3 | no |  |
| **52** | **Stereo** | **3** | **Monica** | **7** | **1** | **BS7** | **2** | **BS8** | **Partner died** |
| 53 | Geralt | 3 | Barbossa | 7 | 1 | BS7 | 3 | BS8 | Partner left |
| 54 | Pirat | 14 | Milagros | 4 | 1 | BS7 | 1 | BS7 | Partner left |
| **55** | **Hachico** | **5** | **Sheloba** | **4** | **2** | **BS8** | **2** | **BS9** | **Unsuccessful BA** |
| 56 | Pierre | 9 | Raya | 4 | 2 | BS8 | 2 | BS9 | Unsuccessful BA |
| **57** | **Stereo** | **4** | **Harper** | **5** | **2** | **BS8** | **4** | **no** |  |
| **58** | **Chowder** | **3** | **Barbossa** | **8** | **2** | **BS8** | **4** | **no** |  |
| 59 | Tappi | 2 | Dolores | 2 | 1 | BS8 | 3 | no |  |
| **60** | **Bruce** | **17** | **Raya** | **6** | **1** | **BS9** | **1** | **no** |  |
| **61** | **Batman** | **2** | **Henia** | **2** | **1** | **BS9** | **2** | **no** |  |
| **62** | **Rambo** | **2** | **Roli** | **2** | **1** | **BS9** | **3** | **no** |  |
| **63** | **Rainbow** | **1,5** | **Wusia** | **2** | **1** | **BS9** | **2** | **no** |  |

**IN BOLD – breeding pairs** / Normal font – non-breeding pairs;

M. Age / F. Age – represents the age of a male / female at their first breeding attempt at Zoo Wrocław;

Bond – represents the time in years that the pair stayed together;

1^st^ BA – represents the breeding season in which the pair had its first breeding attempt;

BAs – represents a number of overall breeding attempts for the pair;

Partner change – represents the breeding season(s) in which a change of partner occurred in the pair;

Change reason – describes the reason for a partner change or is stated as “Unknown”

^a^ – After 6 unsuccessful breeding attempts both changed partners in BS2 and reunited in BS6

^b^ – Pair breeds successfully in all BSs, in BS8 after a successful BA, male changes partner to Sheloba and breeds successfully with her as well, than continues breeding with original partner in next BS

^c^ – Female breeds simultaneously and successfully with two other males

^d^ – Female consistently attempts to breed simultaneously with two males for 3 BSs with no success

^e^ – Pair breeds successfully in early BS8 and the female changes partner later in the same season

**Table S2.** Numbers of individuals and pairs in each breeding season and their overall breeding performance.

| Breeding season | Colony size | Pairs in colony | BA | Eggs | CH | CS | **Clutch size** | **CH/BA** | **Fledged** | **Brood size** |
| --- | --- | --- | --- | --- | --- | --- | --- | --- | --- | --- |
| BS 1 | 55 | 10 | 13 | 27 | 7 | 7 | 2,08 | 0,54 | 0,54 | 1,75 |
| BS 2 | 68 | 20 | 40 | 68 | 19 | 18 | 1,70 | 0,48 | 0,45 | 1,50 |
| BS 3 | 89 | 25 | 53 | 94 | 39 | 26 | 1,77 | 0,74 | 0,49 | 0,93 |
| BS 4 | 112 | 28 | 63 | 115 | 35 | 21 | 1,83 | 0,56 | 0,33 | 1,00 |
| BS 5 | 116 | 30 | 62 | 113 | 15 | 8 | 1,82 | 0,24 | 0,13 | 0,73 |
| BS 6 | 112 | 37 | 95 | 169 | 31 | 13 | 1,78 | 0,33 | 0,14 | 0,62 |
| BS 7 | 125 | 38 | 78 | 148 | 22 | 16 | 1,90 | 0,28 | 0,21 | 1,14 |
| BS 8 | 116 | 35 | 79 | 145 | 26 | 23 | 1,84 | 0,33 | 0,29 | 1,35 |
| BS 9 | 105 | 34 | 68 | 125 | 3 | 2 | 1,84 | 0,04 | 0,03 | 1,00 |

BA - breeding attempts; Eggs - number of eggs laid; CH – number of chicks hatched; CS - number of chicks survived; Fledged – number of chicks fledged


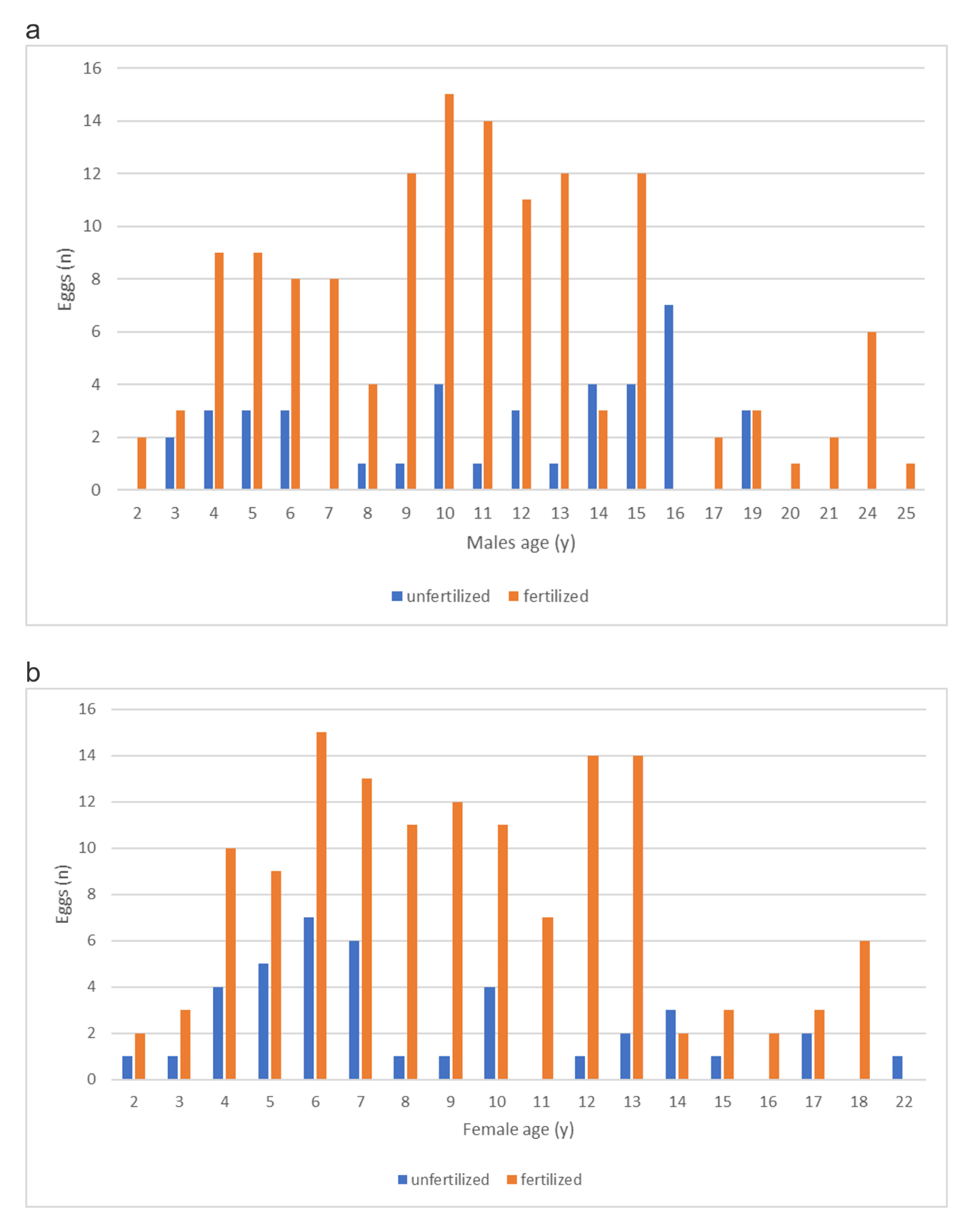


**Figure S1.** Numbers of eggs laid by African penguin pairs with a breeding ban, categorized as fertilized or unfertilized, in relation to the age of males (a) and females (b).
